# A Novel Mutation in a Child with Atypical Wiskott-Aldrich Syndrome Complicated by Cytomegalovirus Infection

**DOI:** 10.4274/tjh.galenos.2018.2018.0187

**Published:** 2019-02-07

**Authors:** Zühre Kaya, Cansu Muluk, Şule Haskoloğlu, Lale Ş. Tufan

**Affiliations:** 1 Gazi University Faculty of Medicine, Department of Pediatrics, Division Pediatric Hematology, Ankara, Turkey; 2 Ankara University Faculty of Medicine, Department of Pediatric Allergy and Immunology, Ankara, Turkey; 3 Ankara University Faculty of Medicine, Department of Forensic Medicine Forensic Genetics Laboratory, Ankara, Turkey

**Keywords:** Wiskott-Aldrich syndrome, Juvenile myelomonocytic leukemia, Cytomegalovirus

## To the Editor,

We present the first described case of a young child with newly diagnosed Wiskott-Aldrich syndrome (WAS) caused by a novel mutation in the WAS gene, c.271C>T(p.Q91X), presenting with juvenile myelomonocytic leukemia (JMML)-like clinical features and cytomegalovirus (CMV) infection.

**Figure 1:**
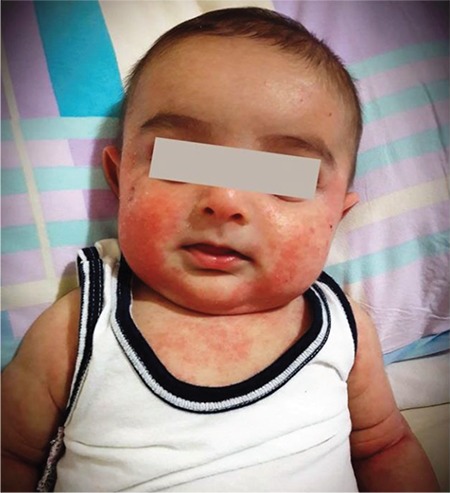
Physical examination revealed widespread eczema.

The proband, a 4-month-old boy, was referred to our hospital for evaluation of eczema, bicytopenia, leukocytosis, and splenomegaly, all of 2 months’ duration. He also had a history of pneumonia. The boy’s parents were not related, and there was a family history of early childhood deaths. Physical examination revealed splenomegaly and widespread eczema, and informed consent was obtained from the parents for Figure 1. Laboratory assessment revealed hemoglobin of 8.4 g/dL and reticulocytes of 1.3%, white blood count of 19,600/mm^3^ (35% eosinophils, 15% monocytes on differential blood count), platelet count of 33,000/mm^3^, and mean platelet volume (MPV) of 10 fL. The direct Coombs test was positive for warm antibodies, and a peripheral blood smear revealed marked eosinophils, monocytes, and immature myeloid cells and giant platelets. Bone marrow examination showed myeloid hyperplasia with eosinophilia. Baseline immunoglobulin (Ig) levels were normal (IgG 317 mg/dL, IgA 14 mg/dL, and IgM 87 mg/dL). The patient was diagnosed with autoimmune hemolytic anemia and had additional diagnostic criteria that suggested JMML and WAS. He was started on prednisone at 1 mg/kg twice daily. Three weeks after initial presentation, he developed shortness of breath, fatigue, and palpitations. He developed a severe pulmonary infection that was successfully treated with trimethoprim-sulfamethoxazole and ganciclovir. A PCR test for CMV was positive, with 9700 copies/mL. Molecular genetic analysis revealed a novel mutation in the WAS gene, c.271C>T(p.Q91X). The patient was diagnosed with WAS. He was scheduled for allogeneic stem cell transplantation from an unrelated donor.

WAS is a rare and potentially fatal disorder of X-linked recessive inheritance that is characterized by recurrent sinopulmonary infections, eczema, and microthrombocytopenia. We report here a young child with newly diagnosed WAS complicated by CMV, with clinical and laboratory findings similar to JMML. Yoshimi et al. reported seven infant boys with WAS who initially presented with leukocytosis, monocytosis, and myeloid and erythroid precursors in their peripheral blood as well as bone marrow dysplasia [1]. The authors noted that the patients’ MPV values were normal or high, which is incompatible with WAS. As we observed in our case, this clinical picture is indistinguishable from JMML. Affected patients may have variable clinical presentations due to disease-modifying genetic factors and different exposure to pathogens [2,3,4]. The causes of JMML-like features in WAS patients are poorly understood. Recent reports suggest that such atypical features may be attributed to coexistence of viral infection or activation of WAS protein by a somatic mutation concomitant with RAS pathway mutations [1,2,3,4,5]. Based on these considerations, we believe that CMV infection was responsible for our patient developing a JMML-like clinical picture and immune cytopenia. Although JMML mutational studies were not performed, persistent monocytosis, splenomegaly, and positive PCR results for CMV all support the diagnosis of CMV infection.

Our experience suggests that physicians should be aware of the potential development of immune cytopenias and JMML-like features in children with WAS who contract CMV infection.
